# Influence of processing parameters on dehydrogenation of TiH_2_ in the preparation of Ti–Nb: A review

**DOI:** 10.1016/j.heliyon.2022.e11602

**Published:** 2022-11-16

**Authors:** Anis Fatehah Sa'aidi, Ahmad Farrahnoor, Hussain Zuhailawati

**Affiliations:** a Centre for Mechanical Engineering Studies, Universiti Teknologi MARA, Cawangan Pulau Pinang, Permatang Pauh Campus, 13500 Pulau Pinang, Malaysia; b Biomaterials Niche Area, School of Materials and Mineral Resources Engineering, Universiti Sains Malaysia, Engineering Campus, Nibong Tebal, Penang, Malaysia

**Keywords:** Implant, Titanium hydride, Dehydrogenation, Biomaterials

## Abstract

Commercially pure titanium (cp-Ti) and Ti–6Al–4V alloy have emerged as excellent candidates for use as biomaterials in medical implants due to their high strength-to-weight ratio and biocompatibility. β-type Ti alloys composed of non-toxic metallic elements such as niobium (Nb) have been extensively studied in order to resolve the issue of a high elastic modulus and toxicity of certain elements, particularly in Ti–6Al–4V alloy. Titanium hydride (TiH_2_) has recently received a lot of attention due to its densification, oxidation levels, and material costs. Powder metallurgy combined with mechanical alloying has become an attractive route for producing near-net shape components of Ti-based alloys, mainly where porosity control and better homogeneity are required. This review aims to create a platform for investigating the feasibility of producing Ti from TiH_2_ via a dehydrogenation process. The dehydrogenation behaviour of TiH_2_ is affected by variables such as sintering condition, alloying element, and particle size. The review revealed that TiH_2_ decomposition occurs at various temperatures (400 °C to 800 °C), resulting in the formation of several sequences of phases. Although the dehydrogenation process was unaffected, the addition of alloying elements was found to change the starting and ending temperatures of the reactions. The use of vacuum accelerates the dehydrogenation process more than argon flow. TiH_2_ powder with smaller particle size, on the other hand, eliminates hydrogen faster than larger ones due to the larger surface area exposed. This review also looks at the best processing conditions for getting a high concentration of β phase in Ti–Nb alloys. β-type titanium alloys with a low elastic modulus (10–40 GPa) similar to human bone are a potential strategy for reducing premature implant failure.

## 1 Introduction to titanium (Ti) and its alloys

Global demand for metallic biomaterials is rising quickly due to an ageing population and the increased risk of hard tissue failure as people get older [1]. Stainless steel (8.0 g/cm^3^) and cobalt-based alloys (8.5 g/cm^3^) have higher densities than titanium (4.54 g/cm^3^), which is more comparable to bone density (1.8–2.0 g/cm^3^) [2, 3]. Therefore, commercially pure titanium (cp-Ti) was initially used to replace 316 L stainless steel and cobalt-chromium (Co–Cr) alloys in the development of bone implants due to its density, as well as superior biocompatibility and corrosion resistance when in contact with human tissues and bodily fluids [4]. However, cp-Ti has the disadvantage of having low mechanical characteristics that led to its substitution with (α + β) type Ti–6Al–4V alloy [5].

**Figure 1:**
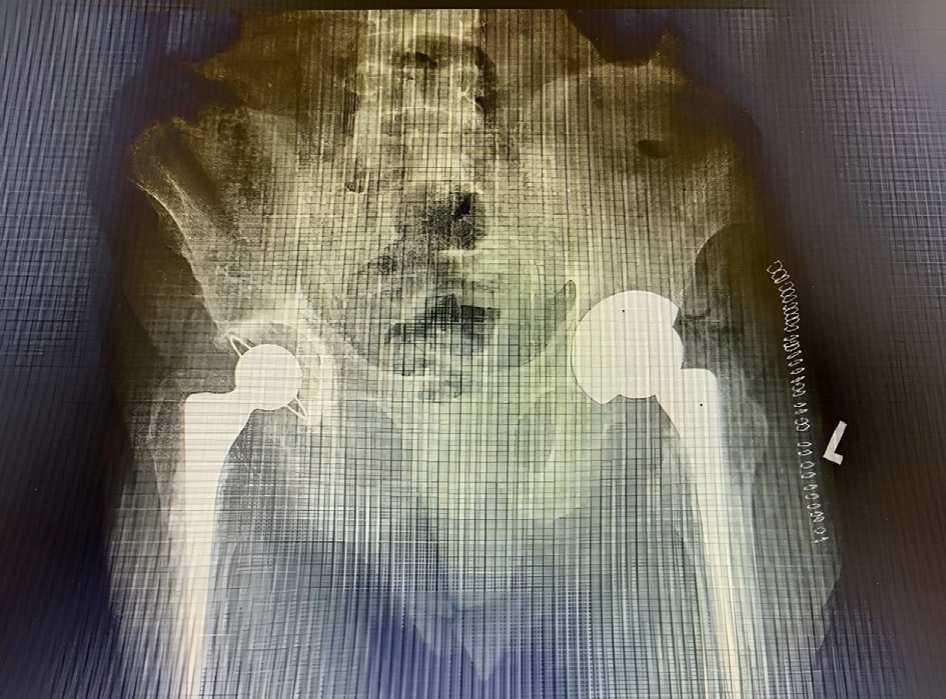
Total hip replacement (THR).

Ti–6Al–4V alloy has been used more frequently in bone implants, particularly for artificial hip joint replacements in patients with hard tissue failure due to its excellent corrosion resistance, high strength, and biocompatibility [6, 7], as shown in Figure 1. Hard tissue replacement can also be accomplished with the use of bone plates and spinal fixation rods. However, the presence of vanadium (V) and aluminium (Al) in Ti–6Al–4V alloy has raised concerns due to their toxicity to the human body. Long-term implantation of aluminium may cause neurological disorders, including Alzheimer's disease, while vanadium may trigger allergic reactions and cytotoxicity [8, 9]. The elastic modulus of Ti–6Al–4V alloy is also greater (100–120 GPa) than that of a human cortical bone (10–40 GPa) [10]. The high modulus of Ti–6Al–4V alloy is caused by the high content of aluminium which increase the amount of α (alpha) phase [11].

Differences in the elastic properties of the implant and human bone result in inhomogeneous stress and strain distributions at the interface between bone and implant. In the areas where the implants are attached to the bone, this may result in poor osseointegration (poor bone-titanium implant contact integration), which can cause crack nucleation and raise the risk of bone fracture. As a result, the patient will be in excruciating pain and a revision surgery would be necessary [12, 13]. A mismatch in elastic modulus should be avoided as much as possible to decrease the stress shielding effect by selecting the most suitable biomaterial with acceptable qualities [14]. Niobium (Nb) has been studied extensively as a potent β phase stabilizer with a high potential for forming a homogeneous β (beta) phase in titanium (Ti) matrices [15, 16]. In order to achieve this objective, significant research is currently being conducted to identify non-toxic Ti alloys with a lower elastic modulus, closer to that of cortical bone, through the production of Ti–Nb alloys (β-Ti-Nb).

The comparatively high cost of Ti and its alloys has limited its applicability in the development of bone implants. This high price is a result of the price of basic production procedures and numerous expensive downstream processing stages required to transform Ti sponge into a useful form. Moreover, low thermal conductivity of Ti and its high oxidation driving force make it difficult to fabricate parts through manufacturing processes such as cutting and forming. As a result, powder metallurgy based on blended elemental powders would be a promising method of fabricating Ti and its alloys because it is a cost-effective technique for producing near net shape products with more efficient material use that provide outstanding properties comparable to wrought materials [17]. In general, β-type Ti alloys contain a high concentration of alloying elements with high melting points (above 1500 °C). Because of the large differences in melting points between Ti and its alloying elements, powder metallurgy can be used to prepare materials with non-equilibrium compositions. This is due to the fact that it can combine different elements in powder form. Another advantage of powder metallurgy is the ability to create interconnected porosity. A porosity between 20% and 50% improves cell-tissue interaction [18]. Powder metallurgy permits the attainment of porosities between 20% and 25% while retaining high mechanical properties [19]. The combination of powder metallurgy and mechanical alloying increases the homogeneity of alloy compositions by combining different elemental powders at the atomic level through rapid welding and fracturing with high-energy ball milling. Numerous studies have discussed the preparation of Ti-based alloys using a combination of the mechanical alloying and powder metallurgy routes, followed by the sintering process [20, 21, 22].

## 2 Advantages of titanium hydride (TiH_2_)

There is a growing interest in using titanium hydride (TiH_2_) as an alternative strategy to obtain Ti powder because it reduces production costs by a factor of nine, resulting in more affordable prices [20]. This requires the hydrogenation–dehydrogenation (HDH) process, in which hydrogen from TiH_2_ is dehydrogenated to produce Ti powder [23]. Consequently, the role of TiH_2_ as a raw material that serves as an intermediate product in the production of Ti by HDH can lead to a reduction in costs.

The hydrogen produced during TiH_2_ decomposition process provides a protective environment for the Ti surface, thereby controlling the amount of contamination [24]. Due to the hydrogen gas produced during dehydrogenation, the use of TiH_2_ can avoid the interaction of oxygen atoms and provide a reducing atmosphere for Ti by creating a clear, non-oxidised Ti surface for effective bonding and accelerating the reduction process [25, 26]. As a result, hydrogen is capable of providing better contamination control.

Furthermore, TiH_2_ improves powder compaction, resulting in higher green density. This is due to the brittle nature of TiH_2_, as opposed to ductile Ti, which allows particles to be fragmented into smaller sizes during pressing, resulting in higher powder compressibility. It is well known that increasing powder compressibility improves green density and powder densification. Zhang et al. [27] demonstrated this by studying the compaction behaviour of TiH_2_–1Al–8V–5Fe (TiH_2_-185) and Ti–1Al–8V–5Fe (Ti-185) alloy powder prepared in a high energy ball mill, assembled into cylindrical pellets by uniaxial pressing at 200, 400, 600, 800, 1000, and 1200 MPa, and finally sintered under vacuum conditions. TiH_2_-185 has higher densification and sintered density than Ti-185, according to their findings. They also stated that the higher chemical activity and alloying element diffusion rate in TiH_2_-185 due to its higher hydrogen content contributed to its stronger compressibility.

During the TiH_2_ → Ti + H_2_ decomposition stage, a high number of crystal lattice defects are produced, which speed up the sintering activity of the powder due to a rise in vacancies. This circumstance increased the mass transfer across interparticle boundaries and activated the diffusion process by decreasing the activation energy. As a result, the number and size of pores shrink. This increases the density of the sintered alloy and speeds up the final product's chemical homogenisation. This method can achieve densities greater than 95%, eliminating the high cost of hot isostatic pressing (HIP) [28]. In the meantime, the Ti powder obtained by the method of HDH is compared with the TiH_2_ powder by a few authors. According to Savvakin et al. [29], cp-Ti produced by sintering TiH_2_ powder demonstrated density 97%–98.5% of theoretical value, while 94.5%–98% were achieved by sintering Ti HDH powder under the same processing conditions. This is consistent with the findings of Dong et al. [30], who found that TiH_2_ had the highest relative density, reaching as high as 88.7% at 600 MPa when compared to Ti HDH, because the final oxygen content in TiH_2_ powders was lower than that in materials processed from Ti HDH powders.

**Figure 2:**
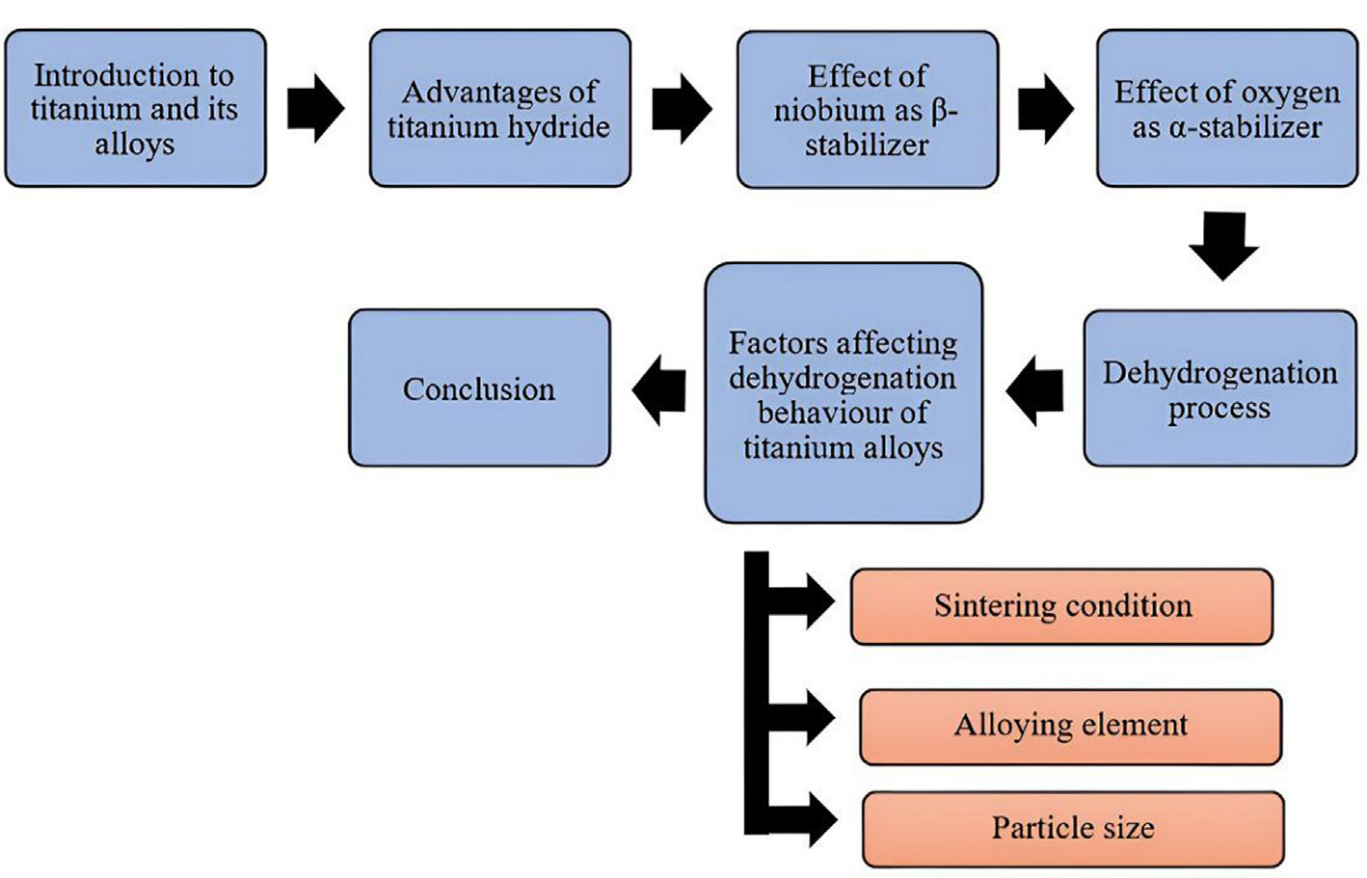
Outline of this review paper.

Existing studies recognise TiH_2_ critical role in the production of Ti-based alloys with good mechanical properties and low elastic modulus [31, 32]. Nonetheless, the relationship between TiH_2_ dehydrogenation behaviour and the formation of β-type Ti alloys is not fully understood. The extent to which various factors influence the TiH_2_ decomposition process is unknown. Thus, the goal of this review paper is to critically examine the benefits of TiH_2_, the effect of Nb as β-stabilizer in Ti-based alloys, the effect of oxygen as α-stabilizer, the dehydrogenation process, and the influence of processing parameters on its behaviour, as shown schematically in Figure 2.

## 3 Effect of niobium (Nb) as β-stabilizer

Ti is divided into α alloys, near-α alloys, α + β alloys, and β alloys [33]. The addition of alloying elements influences the stability of α phases and β phases, resulting in the transformation temperature between the α hexagonal close-packed (α-HCP) and β body-centred cubic (β-BCC) phases [34, 35]. Ti exists in HCP crystal structure at temperatures lower than 882 °C, and this structure is referred as α phase. The hexagonal close-packed (HCP) crystal structure allotropically transforms into a body-centred cubic (BCC) structure, known as the β phase when Ti is heated to a temperature greater than 882 °C [36]. When certain alloying elements are introduced to Ti, they tend to alter the amount of each phase and the β-transus temperature in a predictable manner. The transus temperature is also known as the transition or transformation temperature. The Ti alloy's β-transus temperature is the lowest temperature below which phase equilibrium does not occur. The β phase persists until the temperature reaches 1670 °C, at which point it begins to melt [37]. Regarding near-α alloys, a high proportion of stabilizers and a small amount of β phase are present. The mechanical property of α + β phase alloys is balanced between the two phases, and at room temperature, the phase β amount can range between 10% and 50%.

**Figure 3:**
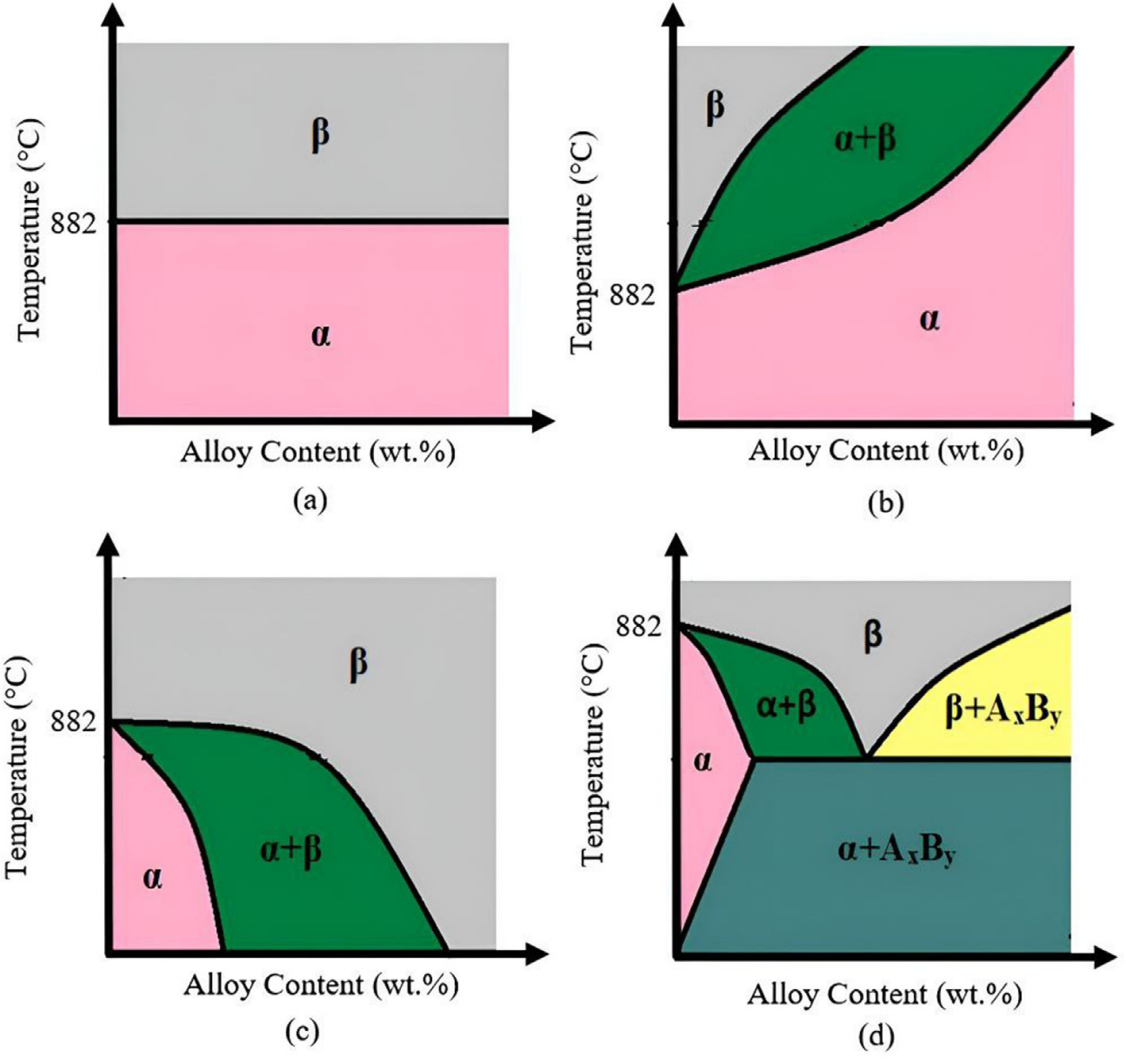
Types of stabilizers in Ti alloys: (a) neutral; (b) α-stabilizing; (c) β-stabilizing (isomorphous), and (d) β-stabilizing (eutectoid).

Figure 3 shows the phase transformation of Ti alloys based on various types of stabilizers. Alloy content in Figure 3 refers to the composition of alloying element added into the Ti matrix. α-stabilizers include elements such as aluminium (Al), zirconium (Zr), oxygen (O), nitrogen (N), and carbon (C). It raises the transformation temperature and performs well at high temperature as shown in Figure 3(b). In general, α and near-α alloys have lower strength and bending ductility than α+β or β alloys; as a result, α and near-α alloys have not been utilised in implant applications. The β phase has been identified in order to produce alloys with a lower elastic modulus (40 GPa–80 GPa) [38]. β-stabilizers can be classified as either isomorphous or eutectoid based on the addition of alloying elements to Ti. Ti is highly soluble in isomorphous elements, such as niobium (Nb), vanadium (V), molybdenum (Mo), and tantalum (Ta) that decrease the transformation temperature as presented in Figure 3(c) [39]. However, Figure 3(d) shows eutectoid elements, such as manganese (Mn), chromium (Cr), silicon (Si), iron (Fe), cobalt (Co), nickel (Ni), and copper (Cu) are insoluble in Ti and are prone to form intermetallic compounds. Meanwhile, neutral elements, such as tin (Sn), hafnium (Hf), and zirconium (Zr) are regarded to have no effect on the phase border as shown in Figure 3(a) [40]. As a result, care should be taken when selecting alloying elements with Ti, particularly in stabilizing the β phase of Ti to a lower temperature according to the binary equilibrium phase diagram.

Incorrect alloying material selection and low β phase stability can lead to serious implant material difficulties. Different alloying elements, such as niobium (Nb), tantalum (Ta) and zirconium (Zr) are fully inert in vitro. However, molybdenum (Mo) is hazardous, making it unsuitable for consideration as a potential biomedical material [41]. Based on these bioinert criteria, Nb should be recognised as the more advantageous alloying element for Ti than the other elements due to the absence of toxic effects in the organism. Aside from that, Nb is known as one of the best β-stabilizers for preventing the formation of α phase. Nb has been used to create binary alloys with Ti known as Ti–Nb alloys, which have two distinct elements that provide superior mechanical biocompatibility [42], corrosion resistance [43], and a low elastic modulus [44], making them an excellent choice for implant applications. As a result, the non-toxic and allergy-free Ti alloyed with Nb has piqued the interest of implant materials researchers in conducting additional research on the various content of Nb that affect a complete and stable phase. Farrahnoor and Zuhailawati [45] demonstrated through their research that 30–45 wt.% Nb resulted in the most ideal composition for producing Ti-based bone implants with higher formation of β phase as an alternative to Ti–6Al–4V alloy. This is consistent with the findings of a study conducted by Chen and Thouas [46], who concluded that Nb concentration increases β phase stability at 40–45 wt.% because it can maintain purely phase at room temperature. Mahran et al. [47] recently chose 34 wt.% Nb to promote β phase stabilization in Ti alloy fabricated using powder metallurgy technique composed of high energy ball milling at consolidation temperatures ranging from 900 °C to 1000 °C. They discovered a low elastic modulus of 63.93 GPa–66.4 GPa, similar to the elastic modulus of cortical bone tissue. Other researchers have found that binary Ti–Nb alloys containing low content of Nb roughly 40-45wt.%, have a low elastic modulus in the range of 50 GPa–60 GPa [48, 49]. Meanwhile, Zhang et al. [50] used a vacuum consumable electrode arc furnace to investigate the effect of low Nb content in Ti-xNb alloys with x = 5, 10, 15, 20, and 25 wt.%. They discovered that Nb content affects β phase formation, with Ti–5Nb having the least amount of β phase and Ti–25Nb having the most. In addition, they suggested that Ti-(10–25 wt.%) Nb exhibits enhanced mechanical properties and comparable biological performance, with Ti–25Nb exhibiting the highest strength (1014 MPa) with a comparable elastic modulus (77.1 MPa). Yilmaz et al. [51] found that the elastic modulus of Ti-xNb (x = 16, 28, and 40 wt.%) alloys decreased from 140 GPa to 100 GPa as the proportion of the β phase increased with the addition of Nb to Ti. They also discovered that the addition of Nb reduced elastic modulus from 140 GPa to 100 GPa due to an increase in β phase, while increasing hardness from 269 HV to 340 HV. As a consequence, the mechanical properties of the load-bearing implant can be significantly altered by varying the content of Nb used.

Nonetheless, alloying TiH_2_ with Nb can affect the phase transformation of Ti alloys. The face-centred cubic (FCC) structure of δ-TiH_2_ was transformed into the HCP structure of α-Ti during the dehydrogenation process. After the diffusion of Nb into its matrix, this structure converted into a β-Ti with the BCC structure. Hosnie et al. [52] investigated the powder metallurgy process for producing porous β-type Ti–40Nb alloys from TiH_2_. TiH_2_ and Nb powders were mixed, compressed, and sintered at 1200 °C in a tube furnace using a heating rate of 5 °C/min under flowing argon gas. TiH_2_ was decomposed in two steps by hydrogen to produce α-Ti. Nb was diffused into Ti matrix after being completely dehydrogenated to form β-type Ti alloys. They discovered that the β-Ti peak in x-ray diffraction (XRD) overlapped with the Nb peak after the sample was sintered. Despite the fact that the results demonstrated that Nb atoms did not diffuse completely into the Ti matrix, β phase was obtained. The absence of a TiH_2_ peak in the XRD patterns led to the assumption that all hydrogen had been decomposed from TiH_2_. The resultant Ti–40Nb alloy with abundant β phase produced a low elastic modulus of 17 GPa and compressive strength of around 300 MPa. Thus, it can be inferred that β-type Ti alloys with a low elastic modulus are a viable technique to promote an effective transfer of mechanical stress, by offering a stress-shielding effect at the interface between the implant and the neighbouring bone, thereby preventing bone cell damage.

## 4 Effect of oxygen (O) as α-stabilizer

Oxygen is a α-stabilizer, which is frequently one of the most effective elements in Ti alloys. At elevated temperatures, high reactivity with the surrounding impurities (such as oxygen) causes an increase in the oxygen concentration of Ti, which significantly affects its phase stability and performance. Oxygen is very soluble in α and β prior to forming oxide phases. The dissolution of oxygen in α and β results in the hardening of interstitial solid solution strengthening and the reduction of ductility [53]. According to Na et al. [54], excess oxygen should be removed because it reduces Ti toughness when the oxygen concentration exceeds 0.3 wt.%. In another study, Wei et al. [55] proposed that the oxygen content in Ti alloy must be less than 1.5% in order to maintain the low modulus at a low level (less than 65 GPa) and be appropriate for biomedical implants. According to Liu et al. [56], oxygen diffusion results in a uniform α-case layer beneath the surfaces of Ti alloy, which has a significant impact on its mechanical properties. Park et al. [57], suggested that dehydrogenation could be used to reduce the oxygen concentration from 0.282 wt.% to 0.216 wt.%. This is due to the fact that hydrogen released from TiH_2_ powder has a cleansing effect on the particle surface, protecting Ti from being exposed to oxygen. Thus, the oxygen content of Ti should be controlled because it has a significant impact on the phase stability of β-Ti alloys. As a result, it is possible that the dehydrogenation process can lower the elastic modulus of Ti alloys by obtaining a higher number of β phases due to the cleaning effect of hydrogen removal, which tends to remove oxygen from the Ti surface.

## 5 Dehydrogenation process

TiH_2_ is commonly used to produce Ti products with acceptable performance via the hydrogenation-dehydrogenation (HDH) process [58]. Ti powders react with a high concentration of hydrogen at high temperatures to produce TiH_2_: Ti(s) + H_2_→ TiH_2_(s) [59]. As an alloying element, hydrogen can be incorporated into the Ti matrix by annealing in either a vacuum or an argon atmosphere [60]. Dehydrogenation occurs faster in vacuum than in argon flow. This is because hydrogen is continuously removed during the decomposition of TiH_2_ when the samples are under vacuum. The hydrogen from TiH_2_ is then removed through dehydrogenation to obtain Ti. During the dehydrogenation process, TiH_2_ releases hydrogen to form pure Ti: TiH_2_(s) → Ti(s) + H_2_, where (s) refers to solid state of the chemical substances [61]. The transformation of Ti when releasing hydrogen is commonly studied to understand the effect of dehydrogenation behaviour on Ti-based alloys.

**Figure 4:**
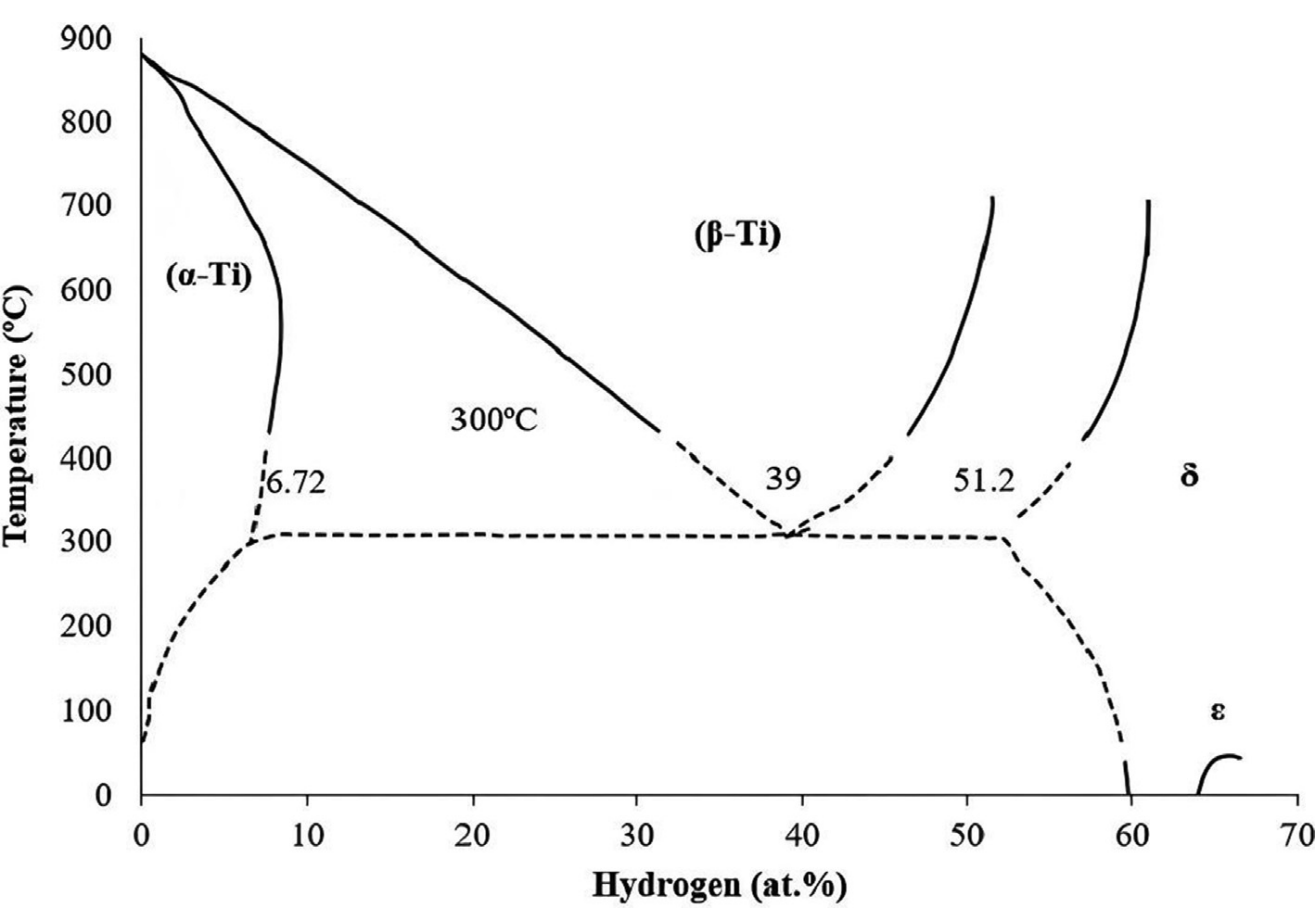
Ti–H phase diagram.

As seen in the Ti–H phase diagram (Figure 4), different allotropic forms of TiH_2_, such as, α, β and δ (delta) can be created by varying the hydrogen content, temperature, and pressure. Titanium hydrogen solubility increases as the temperature rises, and hydrogenation results in the creation of δ-hydride. Phase transitions occur in the sequence of α+β phase, β phase, β+δ phase and δ phase during hydrogenation above 300 °C. After cooling to room temperature from any of the aforementioned phase areas, it transitions into α+δ-phase [62]. As a result, the dehydrogenation behaviour of TiH_2_ can influence phase transformation of Ti alloys.

The most common method of using TiH_2_ for most applications is dehydrogenation to form α-Ti via heat treatment in a controlled atmosphere. The presence of distinct δ-hydride phases in the Ti–H phase diagram indicates the possibility of metastable or stable hydrides during dehydrogenation [63]. TiH_2_ metastable phase and dehydrogenation behaviour can be studied using Rietveld refinement and high temperature XRD [64]. Aside from that, differential thermal analysis (DTA) and thermogravimetric analysis (TGA) are frequently used to understand TiH_2_ thermal and decomposition behaviour. Depending on the temperature and duration of dehydrogenation, the TiH_2_ phase sequence can shift from δ-TiH_2_ to β phase and then back to α phase, with the metastable phase in between. These changes result in titanium hydrogen solubility, also known as hydrogen diffusion. Suwarno et al. [65] investigated the transformation of δ-TiH_2_ into α-TiH_X_ after dehydrogenation. According to the results, hydrogen decomposition took two steps, as two peaks were observed. The hydrogen desorption process in vacuum and gaseous flow yielded δ−TiH_1.9_ → β−TiH_1.9-X_ → α−TiH_X_. This is consistent with the findings of Jang et al. [66], who described the dehydrogenation process as two steps: TiH_2_ conversion into TiH_X_, followed by TiH_X_ decomposition into Ti and H_2_. To investigate the dehydrogenation process, Ti tuning chips were subjected to the HDH process to produce Ti from hydrogenated TiH_2_. According to the DTA curves, as milling time increased, the peaks for both reactions shifted toward lower temperatures, and their activation energies decreased. In addition, they reported that the conversion of TiH_2_ to TiH_X_ did not take place in unmilled TiH_2_ and that the activation energy for the breakdown of TiH_2_ to Ti and hydrogen was as high as 929 kJ/mol. Sharma et al. [67] recently investigated the powder metallurgy route for Ti–40Nb alloy fabrication using TiH_2_. TiH_2_ and Nb powders were mechanically alloyed at 200 rpm for varying periods of time before being consolidated using the Spark Plasma Sintering (SPS) method under 50 MPa pressure and high vacuum conditions. TiH_2_ dehydrogenation occurred in two stages, according to the DTA peak and weight loss in the TGA profile. At 297 °C, partial hydrogen decomposition began to form TiH_X_, while complete hydrogen elimination occurred at temperatures greater than 950 °C, resulting in the α-Ti phase. Liu et al. [68] investigated the non-isothermal dehydrogenation of TiH_2_ using TGA and differential scanning calorimetry (DSC). They proposed that the dehydrogenation process be divided into four steps:

(1) δ(TiH_2_) → δ(TiH_X_) → β(Ti)_H_ → β(Ti)_H_ + α(Ti)_H_ → α(Ti)

where β(Ti)_H_ and α(Ti)_H_ in Eq. (1) represented H_2_ rich β phase and α phase. Four stages occurred during H_2_ decomposition process to produce a complete α-Ti.

Numerous authors concur on only the initial stage of the breakdown, which results in the progressive loss of hydrogen while preserving the FCC structure. Another theory suggests that the phase structure of TiH_2_ decomposition changed from FCC to HCP. Liu et al. [68] proposed a four-stage dehydrogenation process in which hydrogen atoms are liberated and their positions would randomly change from tetrahedral to octahedral interstitials sites during the first stage, while the fundamental FCC structure of δ-TiH_2_ is maintained. As hydrogen is lost during the second stage, the δ-TiH_2_ phase would eventually transform into a BCC-structured, hydrogen-rich β-Ti_H_ phase. In the third stage, H_2_ atoms are continuously released from β-Ti_H_, and α-Ti_H_ would appear when the H_2_ count is low enough. According to their findings, stages II and III produced up to 80% dehydrogenation of TiH_2_. Frequently rapid and intense, these stages occurred between 500 °C and 700 °C. The phase transition model had a core-shell structure with a nucleus of δ-TiHx, an intermediate layer of β-Ti_H_, and an outer layer of α-Ti_H_. Finally, complete hydrogen removal was achieved through total α-Ti transformation. In this case, the phase structure shifted from FCC to HCP. Chirico et al. [69] confirmed this observation by demonstrating that the FCC structure of δ-TiH_2_ could be preserved after the first dehydrogenation stage. In their study, TiH_2_ was mechanically alloyed with Nb and Fe before being sintered at 10 °C/min to a temperature of 1250 °C TiH_2_ dehydrogenated in four stages, which was similar to the findings of Liu et al. [68]. During the first stage, partial hydrogen elimination occurred when δ-TiH_2_ transformed into δ-TiH_X_ while retaining its FCC structure at temperatures ranging from 350 °C to 450 °C. Thus, the FCC structure of δ-Ti alloys can be maintained during the initial stage of dehydrogenation and the HCP structure of α-Ti will be obtained once complete hydrogen removal is achieved. Meanwhile, Novoselov et al. [70] reported that the diffusion of hydrogen in TiH_2_ occurred from the first stage. TiH_2_ was sintered at a temperature ranging from 327 °C to 1327 °C to initiate the first dehydrogenation stage. TiH_2_ partially decomposed into δ-TiHx from δ-TiH_2_ phase. Similarly, Hosnie et al. [71] discovered that the FCC structure was preserved after TiH_2_ went through the first stage of hydrogen elimination, where δ-TiH_2_ transformed into δ-TiH_X_. However, only two stages of dehydrogenation were observed in their study. At the end of the procedure, the FCC structure of δ-TiHx was converted into the HCP structure of α-Ti. TiH_2_ goes through several stages before becoming purely α-Ti powder.

Several studies have reported the discovery of α-Ti peaks in XRD patterns, which have been used to demonstrate a complete dehydrogenation process. Mandrino et al. [72] discovered α-Ti peaks in XRD spectra taken from TiH_2_ samples sintered at 800 °C. Jimoh et al. [73] also looked for α-Ti peaks in XRD patterns as TiH_2_ dehydrogenated completely in 2 h at 680 °C. Similarly, Cho et al. [74] found that TiH_2_ completely transformed into α-Ti after being sintered at 650 °C in 1 h. According to the preceding discussion, TiH_2_ decomposition occurs at various temperatures. Accurate temperature of dehydrogenation and phase sequences discovered in the formation of Ti from TiH_2_ can differ. Besides, variety of factors such as sintering condition, alloying element, and particle size must also be taken into account in order to promote decomposition of TiH_2_.

### 5.1 Effect of sintering condition

**Figure 5:**
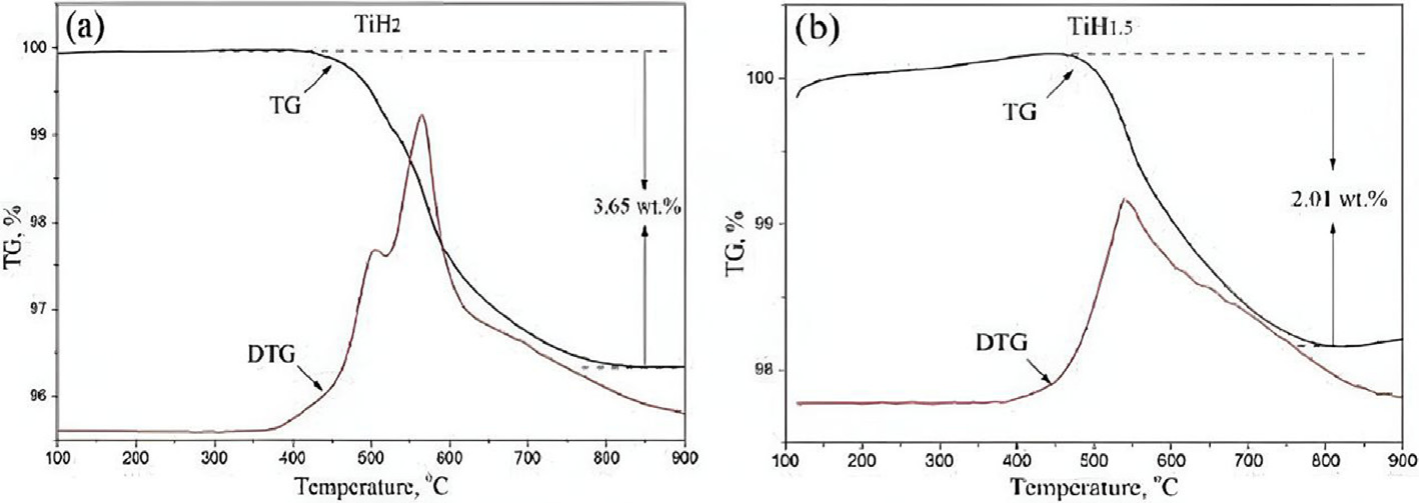
TG curves of (a) TiH_2_ and (b) TiH_1.5_. Reprinted with permission from Elsevier [77].

Hydrogen removal from TiH_2_ commonly occur during sintering. When TiH_2_ is sintered at a high temperature, hydrogen is released from the alloys is considered as dehydrogenation process. The dehydrogenation behaviour of TiH_2_ can be influenced by the sintering parameters, such as temperature, heating rates, duration, and sintering environment. According to Sandim et al. [75], δ-TiH_2_ was converted into α-Ti where the phase structure of the metal was altered from FCC to HCP. This transformation was measured in a high vacuum chamber at different temperatures of 450, 500, 550, and 650 °C, and a heating rate of 30 °C/min. Only δ phase peaks were discovered at temperatures lower than 500 °C. The presence of the α phase was detected at temperatures greater than 550 °C. When the temperature was raised even higher, the TiH_2_ became completely dehydrogenated. It was assumed that the kinetics of hydrogen desorption were faster and completed in less than 50 min. Thus, dehydrogenation can be affected by varying the sintering temperature. Jeon et al. [76] have also reported that TiH_2_ thermally decomposed at approximately 600 °C to form Ti. According to their studies, TiH_2_ and polymethylmethacrylates (PMMA) beads were mixed and sintered for 2 h at 1100 °C with a rate of 10 °C/min to fabricate a porous Ti. Based on the TGA curves, the relative weight of TiH_2_ began to decrease as the temperature approached 600 °C due to the release of hydrogen during the dehydrogenation of TiH_2_ into Ti. Mei et al. [77] investigated different sintering temperatures to identify their effect on hydrogen content in TiH_2_ through powder metallurgy. HDH-Ti, TiH_1.5_, and TiH_2_ were moulded and heated separately at 800, 900, 1000, 1100, 1200, and 1300 °C. They found that TiH_1.5_ and TiH_2_ released hydrogen gas during the sintering process, starting from 400 °C to 850 °C. Elimination of hydrogen can be seen in the TG (thermogravimetric analysis) results as shown in Figure 5, where hydrogen content of TiH_1.5_(b) and TiH_2_(a) 2.01 wt.% and 3.65 wt.%, respectively. When XRD patterns revealed the presence of α-Ti peaks, complete dehydrogenation was achieved. Meanwhile, Ivanova et al. [78] reported that fine and active Ti particles were formed by the dissolution of TiH_2_, led by the rapid establishment and development of interparticle interactions at low sintering temperatures that ranged from 780 °C to 800 °C. The densification of Ti was increased by several times at high sintering temperatures, obstructing the removal of contaminants from its pores, while drastically enhancing its strength and diminishing its ductility.

**Figure 6:**
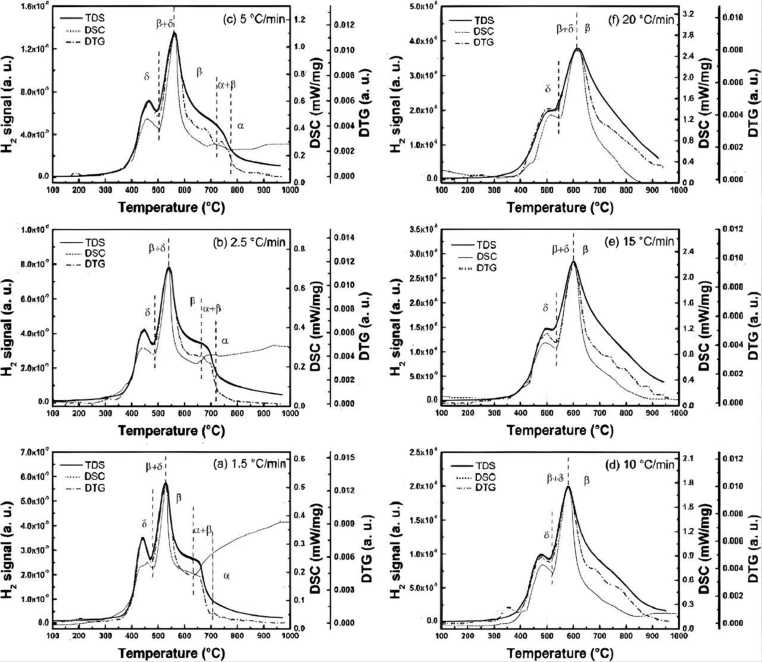
TDS spectra, DSC and DTG curves of as received TiH_2_ at different heating rates. Reprinted with permission from Elsevier [80].

Several studies have reported that dehydrogenation behaviour can be controlled by using different heating rates. Sandim et al. [75] investigated the thermal decomposition of TiH_2_ using different heating rates of 5, 10, and 20 °C/min. It was discovered that the temperature at which hydrogen began to leak was dependent on the heating rate. When the heating rate was decreased, the onset temperature also decreased, according to the findings. The final concentration of hydrogen in the converted powder was, as expected, affected by the heating rate. Reduced heating rates allowed for longer residence times, allowing for greater thermal decomposition. Rasooli et al. [79] also stated that increasing the heating rate would shorten the time required for oxygen atoms to diffuse within the surface layers. They reported that less titanium oxide particles had formed on the surface of the particles, leading to an increase in powder weight. The results showed that the final weight percent increased from 51 wt.% until 58 wt.% and then, reduced to 44 wt.% when the heating rate was increased from 5 °C/min to 10 °C/min and from 10 °C/min to 30 °C/min, respectively. In another study, Ma et al. [80] investigated TiH_2_ phase transition in a thermal desorption process at different heating rates. As shown in Figure 6, the thermal desorption behaviours of TiH_2_ were examined using thermogravimetric analysis-thermal desorption spectroscopy measurements (TG-TDS) and DSC observations at heating rates ranging from 1.5 °C/min to 20 °C/min. They discovered that the TDS, DTG (derivative thermogravimetry), and DSC curves had a strong temperature correlation, allowing for a simultaneous correlation of H_2_ desorption, mass loss, and heat absorption in TiH_2_. These findings suggested that at a heating rate of lower than 10 °C/min, the sequence of phase transformation can be schematised as follows: δ → β + δ → β → α + β → α. Additionally, three phase zones were cycled through at heating rates equal to or greater than 10 °C/min, which can be outlined as follows: δ → β + δ → β. This study is in line with Wang et al. [81] and Yuan et al. [82] who also proposed 10 °C/min as a suitable heating rate in sintering Ti alloy.

In a study by Li et al. [83], Ti-55 Ti alloy sheet (Ti-5.5Al-3.5Sn-3.0Zr-1.5Mo-0.4Ta-0.4Nb-0.3Si, wt.%) was sintered at different soaking time to investigate the complete dehydrogenation process. The sample was first hydrogenated at 750 °C for 1 h by holding in a pure hydrogen environment before air-cooling to room temperature. Then, the hydrogenated sample was sintered at 700 °C for 0, 0.5, and 2 h. The remaining hydrogen content after dehydrogenation depends on the sintering time. The sample sintered at 2 h was regarded as complete hydrogen elimination due to the content of hydrogen which was is 0.0046 wt.%, lower than the safe usage standard of 0.0125 wt.%. After the 2 h holding time, the grain boundary of β phase was completely broken into smaller flakes that were uniformly distributed, in addition to complete hydrogen elimination. Nonetheless, incomplete hydrogen decomposition occurred between 0 h and 0.5 h of sintering. Meanwhile, Kadoi et al. [84] reported that increasing the sintering time by heating TiH_2_ at 400 °C for 1.5, 3, and 12 h raised the temperature of the initial hydrogen elimination and decreased the amount of evolved gas during reheating. Dehydrogenation, according to Gökelma et al. [85], should be performed under higher vacuum conditions, for a longer period of time, and with an optimised particle size range. They elaborated on the incomplete dehydrogenation process caused by a poor vacuum setup, in which peaks of TiH_1.5_ were observed in Ti–6Al–4V XRD patterns even after the process was completed. Wu et al. [86] investigated the release of hydrogen from TiH_2_ during the fabrication of NiTi. They used thermal analysis and real-time pressure monitoring to investigate the dehydrogenation process in argon, air, and vacuum environments. They discovered that at lower temperatures, the rate of hydrogen freed during the heating process was initially slow, but that at temperatures above 550 °C, the rate of TiH_2_ decomposition increased rapidly. The surface oxide layer could have hampered hydrogen escape measurements taken in both air and vacuum. Because oxygen may react with Ti to form a thin oxide layer on the surface of the particles, hydrogen may be prevented from escaping until the process's temperature or pressure is changed. The presence of a significant amount of hydrogen on the sample reduced their mechanical properties. As a result, understanding the decomposition mechanism is critical for increasing component life.

### 5.2 Effect of alloying element

The dehydrogenation behaviour of Ti alloy can also be affected by the addition of alloying elements. According to Chirico et al. [69], the addition of iron and Nb changed the temperature of the first and end stages of dehydrogenation, in comparison to pure/unalloyed TiH_2_. The decomposition of TiH_2_ was accelerated by Fe and Nb, which reduced the temperature of the first stage by 50–95 °C. Fe and Nb may have also acted as a barrier to the elimination of the remaining hydrogen content in the Ti phase, which delayed the third stage offset temperature by 15–50 °C. The phase transformation of Ti alloy was modified by the addition of Fe and Nb, as the β-transus temperature was reduced. TiH_2_ commonly decomposes as a result of the δ-TiH_X_ transition to α-Ti on the particle surface, whereby the phase structure would switch from FCC to HCP in this situation. However, BCC phase was obtained from the transition of δ-TiH_X_ to β-Ti in the presence of Fe and Nb.

Several studies have revealed that TiH_2_ commonly dehydrogenates between 300 °C and 800 °C [87, 88]. In contrast, the presence of alloying elements was found to accelerate the initial temperature for the decomposition of TiH_2_. Hosnie et al. [71] investigated the thermal properties of porous β-TiNb integrated with TiH_2_ powder. According to their research's DTA curves, the initial hydrogen decomposition of TiH_2_ occurred at 300 °C, as a small endothermic curve with an onset temperature of 350 °C occurred. The initial temperature for this procedure was lower than the initial temperature for the decomposition of unalloyed TiH_2_. This result was consistent with the findings of Chirico et al. [89], who reported that the addition of Fe and Nb decreased the temperature during the initial stage of dehydrogenation. They noted that unalloyed TiH_2_ began to release hydrogen at 510 °C, whereas alloyed TiH_2_ dehydrogenated at temperatures between 465 °C and 503 °C.

### 5.3 Effect of particle size

The particle size of TiH_2_ can also affect the removal of hydrogen from Ti alloys. When the particle size decreases, a larger surface area is exposed to the dehydrogenation process. The effect of different particle sizes of TiH_2_ on dehydrogenation was investigated by Bhosle et al. [90] using thermal analysis and structural investigations. The two-step dehydrogenation process can be expressed as follows:

(2) TiH_2_ → TiH_X_ → α -Ti, where 0.7 < x < 1.1

where x in Eq. (2) referred to hydrogen content. TiH_2_ was partially dehydrogenated to generate the lower hydrogen-containing phase TiH_X_, which was more thermally stable and had a lower hydrogen content. Then the second stage of dehydrogenation occurred, resulting in α-Ti.

Guo et al. [91] investigated the effect of different TiH_2_ particle sizes on metal foam pore structure. TiH_2_ particles with particle sizes ranging from 48 μm to 75 μm, 38 μm–48 μm, 25 μm–38 μm, and less than 25 μm were heated at specific temperatures to release hydrogen. TiH_2_ with the smallest particle size (less than 25 μm) began releasing hydrogen at around 460 °C, while powder with the largest particle size (48–75 μm) began releasing hydrogen at around 520 °C. It can be concluded that the faster the dehydrogenation process begins, the smaller the particle size of the powder. The purpose of using metal foam is to observe the different sizes of pores formed after TiH_2_ dehydrogenation. Hydrogen was released during the heating process, and pores can be obtained from metal foam. Similarly, Sharma et al. [92] reported that TiH_2_ with a small particle size and a larger surface area could achieve a higher hydrogen decomposition percentage. Mechanical milling was used to create Ti-25-Nb-11Sn alloy from TiH_2_, Nb, and Sn, which was then consolidated using Spark Plasma Sintering (SPS) at two different milling times (72 ks and 180 ks). Dehydrogenation began and ended at a lower temperature with a longer milling time based on the TGA results. Peillon et al. [93] discovered that particle size influences TiH_2_ decomposition rate. They reported that TiH_2_ with grain sizes ranging from 5 μm to 20 μm began to release hydrogen at similar temperatures ranging from 350 °C to 400 °C with varying dehydrogenation rates. The hydrogen decomposition rates were 15.5 mg/min for the 5 μm powder and 13.5 mg/min for the 20 μm powder, respectively. Because of differences in surface area, the particle size had an effect on the dehydrogenation rate. As a result, the smaller the grain size of TiH_2_, the higher the rate of dehydrogenation.

## 6 Conclusion

This paper provided an overview of the dehydrogenation behaviour of TiH_2_ powders and their phase transformation in the development of Ti–Nb alloys. The alloy has good mechanical properties, a low elastic modulus, and is biocompatible with human bones. In comparison to Ti, TiH_2_ can improve sintered density, reduce oxygen levels, and lower raw material costs. TiH_2_ is dehydrogenated to remove hydrogen and form pure Ti via the HDH process, which consists of several steps. The most important findings of this review are that different sintering conditions, alloying elements, and powder particle size all affect the decomposition of TiH_2_. To fabricate Ti–Nb alloy from TiH_2_ using powder metallurgy, the TiH_2_ and Nb mixture must be sintered in a vacuum furnace. Understanding the interactions between hydrogen and alloying elements during and after the dehydrogenation process could be an effective tool for enhancing Ti alloy characteristics, particularly in the production of β-Ti alloys.

## Declarations

### Author contribution statement

All authors listed have significantly contributed to the development and the writing of this article.

### Funding statement

This work was supported by the Ministry of Higher Education Malaysia (FRGS/1/2021/TK0/UITM/02/35) ​and Universiti Teknologi MARA.

### Data availability statement

Data included in article/supplementary material/referenced in article.

### Declaration of interests statement

The authors declare no conflict of interest.

### Additional information

No additional information is available for this paper.
